# Optimal cut-offs of depression screening tools during the COVID-19 pandemic: a systematic review

**DOI:** 10.1186/s12888-023-05455-8

**Published:** 2023-12-19

**Authors:** Jieru Zhou, Maja R. Radojčić, Claire E. Ashton-James, Hanqiao Yang, Ziyi Chen, Ruijia Wang, Ying Yang, Jinhua Si, Liang Yao, Ge Li, Lingxiao Chen

**Affiliations:** 1 School of Chinese Materia Medica, Tianjin University of Traditional Chinese Medicine, Jinghai, Tianjin, 301617 People’s Republic of China; 2 Division of Psychology and Mental Health, Faculty of Biology, Medicine and Health, University of Manchester, Manchester, UK; 3 Sydney Medical School, Faculty of Medicine and Health, University of Sydney, Sydney, NSW Australia; 4 West China School of Basic Medical Sciences & Forensic Medicine, Sichuan University, Chengdu, Sichua 610041 People’s Republic of China; 5 Clinical Pharmacokinetics Laboratory, School of Basic Medicine and Clinical Pharmacy, China Pharmaceutical University, Nanjing, 211198 People’s Republic of China; 6 Tianjin University of Traditional Chinese Medicine Library, Tianjin University of Traditional Chinese Medicine, Jinghai, Tianjin, 301617 People’s Republic of China; 7 Department of Health Research Methods, Evidence, and Impact, McMaster University, Hamilton, Canada; 8 School of Public Health, Tianjin University of Traditional Chinese Medicine, Jinghai, Tianjin, 301617 People’s Republic of China; 9 Department of Orthopaedics, Qilu Hospital of Shandong University, Shandong University Centre for Orthopaedics, Advanced Medical Research Institute, Cheeloo College of Medicine, Shandong University, Jinan, Shandong 250012 People’s Republic of China; 10 Department of Biostatistics, School of Public Health, Cheeloo College of Medicine, Shandong University, Jinan, Shandong 250012 People’s Republic of China; 11 Sydney Musculoskeletal Health, School of Health Science, Faculty of Medicine and Health, University of Sydney, Sydney, NSW Australia

**Keywords:** Depression, Optimal cut-off, Covid-19, Systematic review

## Abstract

**Background:**

Studies have reported an increase in the prevalence of depression during the COVID-19 pandemic. The accuracy of screening tools may change with the prevalence and distribution of a disease in a population or sample: the “Spectrum Effect”.

**Methods:**

First, we selected commonly used screening tools and developed search strategies for the inclusion of original studies during the pandemic. Second, we searched PsycINFO, EMBASE, and MEDLINE from March 2020 to September 2022 to obtain original studies that investigated the accuracy of depression screening tools during the pandemic. We then searched these databases to identify meta-analyses summarizing the accuracy of these tools conducted before the pandemic and compared the optimal cut-offs for depression screening tools during the pandemic with those before.

**Result:**

Four original studies evaluating the optimal cut-offs for four screening tools (Beck Depression Inventory [BDI-II], Hospital Anxiety and Depression Scale-Depression [HADS-D], Patient Health Questionnaire-9 [PHQ-9], and Geriatric Depression Scale-4 [GDS-4]) were published during the pandemic. Four meta-analyses summarizing these tools before the pandemic. We found that the optimal cut-off of BDI-II was 14 during the pandemic (23.8% depression prevalence, screening patients with Type 2 diabetes) and 14.5 before the pandemic (17.6% depression prevalence, screening psychiatric, primary care, and healthy populations); HADS-D was 10 during the pandemic (23.8% depression prevalence, screening patients with type 2 diabetes) and 7 before the pandemic (15.0% depression prevalence, screening medically ill patients); PHQ-9 was 11 during the pandemic (14.5% depression prevalence, screening university students) and 8 before the pandemic (10.9% depression prevalence, screening the unrestricted population), and GDS-4 was 1.8 during the pandemic (29.0% depression prevalence, screening adults seen in a memory clinic setting) and 3 before the pandemic (18.5% depression prevalence, screening older adults).

**Conclusion:**

The optimal cut-off for different screening tools may be sensitive to changes in study populations and reference standards. And potential spectrum effects that should be considered in post-COVID time which aiming to improve diagnostic accuracy.

**Supplementary Information:**

The online version contains supplementary material available at 10.1186/s12888-023-05455-8.

## Background

Current guidelines, such as the United States Preventive Services Task Force and the United Kingdom National Institute for Health and Care Excellence, recommend depression screening to improve the early diagnosis and treatment of depression [1–6]. For that purpose, clinicians use validated depression screening tools such as the Patient Health Questionnaire-9 (PHQ-9), Hospital Anxiety and Depression Scale (HADS), Beck Depression Inventory (BDI), Center for Epidemiologic Studies Depression (CES-D), Edinburgh Postnatal Depression Scale (EPDS), and Geriatric Depression Scale (GDS) to identify those with and without depression based on established cut-offs [7–15]. Initial development and validation of these screening tools were based on the false positive rate (the probability that an individual with no disease has a positive test result) and false negative rate (the probability that an individual with the disease has a negative test result) of each cut-off [16–22]. Further studies assessed the sensitivity (the probability that an individual with the disease has a positive test result) and specificity (the probability that an individual without the disease has a negative test result) for each cut-off and reported that the cut-off corresponding to the largest sum of the two was the optimal cut-off [23–27]. The accuracy of the screening tools, including their sensitivity and specificity, can change as the prevalence and distribution of a disease alter in a population or sample-a phenomenon known as the “spectrum effect” [28, 29]. When the cut-off was constant, prevalence decreased due to a decrease in the mean of the underlying trait or the true underlying risk of the disease, resulting in increased specificity and decreased sensitivity [28]. For example, in secondary care, the use of CA125 in the diagnosis of ovarian cancer had a sensitivity of 0.80 with a specificity of 0.75 [30], and when it was used in the primary care, as the prevalence decreased, the sensitivity decreased slightly, to 0.77, and the specificity increased, to 0.94 [31].

Several studies indicate that the prevalence of depressive symptoms increased substantially during the COVID-19 pandemic and may continue to be rising in post-pandemic years due to concerns about the raised cost of life, warfare, and interruptions in food supplies [32–35]. Therefore, the sensitivity and specificity of previously validated screening tools may be affected, as it is unclear whether the optimal cut-offs of these tools are sensitive to changes in depression prevalence. The current study investigated whether the optimal cut-offs of depression screening tools changed during the pandemic. We hypothesized that the increased prevalence of depression caused by the COVID-19 pandemic would change the optimal cut-off for depression screening tools.

## Methods

### Protocol and registration

This systematic review was registered with PROSPERO (CRD42022350324). Results were reported under the Preferred Reporting Items for Systematic Review and Meta-Analysis of Diagnostic Test Accuracy Studies (PRISMA-DTA) [36]. The systematic review team consisted of one medical librarian, one psychological scientist, four epidemiologists, and five medical students.

### Eligibility criteria, search strategy, and study selection

**Fig. 1:**
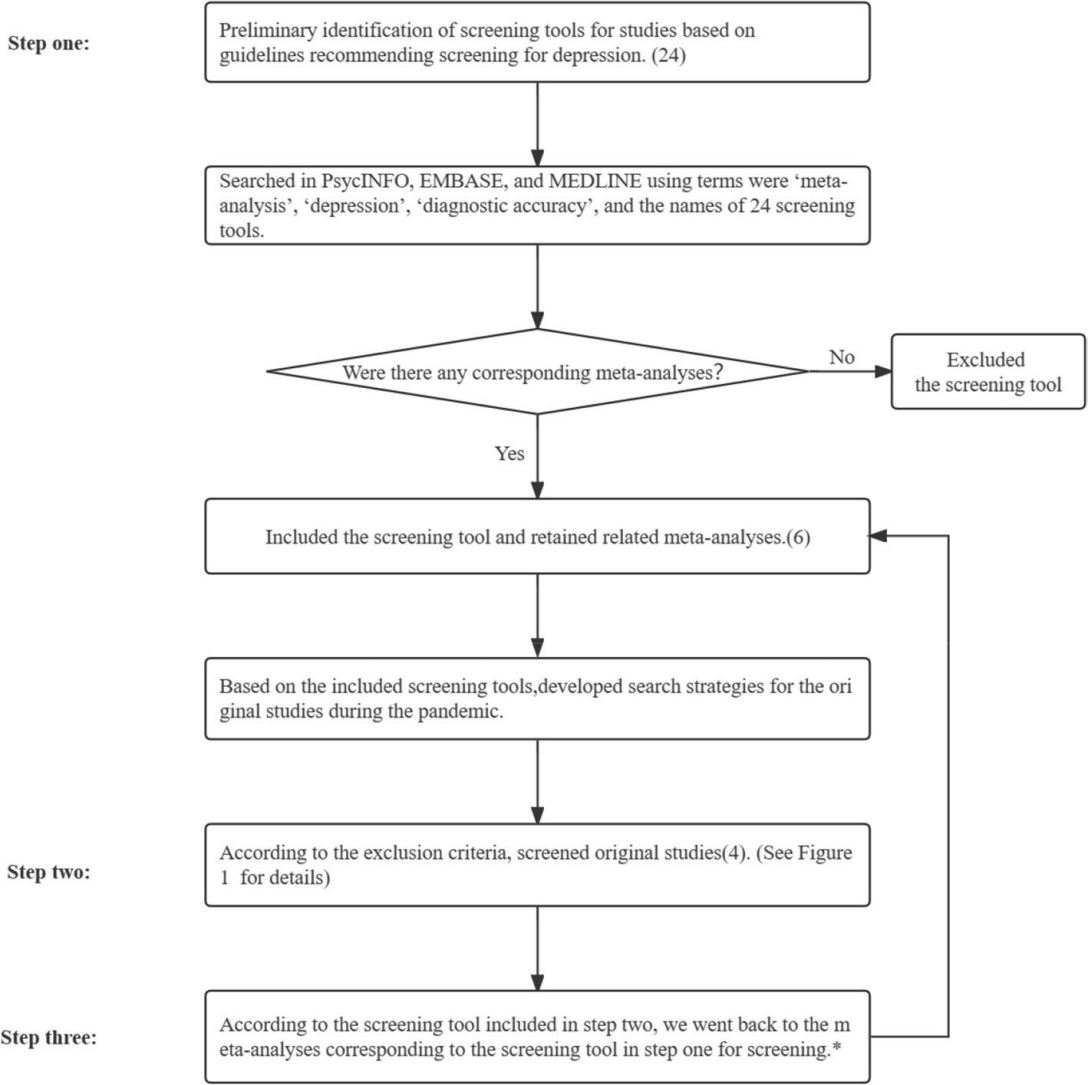
Flowchart of three steps ^*^Taking PHQ-9 as an example, in step two, we included the original study related to PHQ-9 and then screened the meta-analyses related to PHQ-9 retained in step one. The best meta-analysis was selected based on the version of PHQ-9 used in the original study, the population studied, the type of reference standards, and the study quality of the meta-analysis

We included two types of studies and used a three-step method: in step one, we selected commonly used screening tools and developed search strategies for the inclusion of original studies during the pandemic; in step two, during the pandemic, we searched for original studies which focused on the diagnostic accuracy of selected screening tools in step one; in step three, according to the results of step two, we went back to the meta-analyses retrieved in the step one for screening. Figure 1 shows the flowchart of the three steps. The detailed methods were described as follows.

- **Step one: Screening tools and search strategies**

We preliminarily considered 24 screening tools based on 15 guidelines, such as the American Psychological Association (APA), the Royal Australian and New Zealand College of Psychiatrists Clinical Practice (PANZCP), the United Kingdom National Institute for Health and Care Excellence (NICE), and the United States Preventive Services Task Force (USPSTF) [1, 2, 5, 6, 37, 38]. Details are in Supplementary A.

The 24 screening tools were: Patient Health Questionnaire (PHQ), Center for Epidemiologic Studies Depression Scale (CESD), Geriatric Depression Scale (GDS), Edinburgh Postnatal Depression Scale (EPDS), Beck Depression Inventory (BDI), Hamilton Depression Rating Scale (HAM-D), Postpartum Depression Screening Scale (PDSS), Hospital and Anxiety Depression Scale (HADS), Cardiac Depression Scale (CDS), Moods and Feelings Questionnaire, Short Form (MFQ-SF), EuroQol Five Dimensions Questionnaire (EQ-5D), Montgomery-Åsberg Depression Rating Scale (MADRS), Social Problem-Solving Inventory-Revised (SPSI-RTM), Behavior Assessment System for Children (BASC), Child Behavior Checklist (CBCL), Children’s Depression Inventory (CDI), Children’s Depression Rating Scale (CDRS), Beck Hopelessness Scale (BHS), Quick Inventory of Depressive Symptomatology-Self-Report (QIDS-SR), Reminiscence Functions Scale (RFS), Short Form Health Survey (SF-36), Social Adjustment Scale-Self Report (SAS-SR)™, Social Functioning Questionnaire (SFQ), Life Satisfaction Index. Details are in Supplementary B.

Considering that meta-analysis could synthesize all previous studies and provide more accurate information, we investigated whether the diagnostic accuracy of each screening tool had been verified by meta-analyses conducted before the pandemic to determine which screening tools we would finally include. Therefore, we searched PsycINFO, EMBASE, and MEDLINE from inception to 2022 (When we retrieved the three databases) to identify corresponding meta-analyses before the pandemic that summarized the accuracy of depression screening tools. The search terms were ‘meta-analysis’, ‘depression’, ‘diagnostic accuracy’, and the names of 24 screening tools. It was found that only PHQ-9, HADS, BDI, CESD, EPDS, and GDS had their diagnostic accuracy verified by meta-analyses before the pandemic. So, we ultimately decided to use the six screening tools mentioned above to develop search strategies for the inclusion of original studies during the pandemic.

- **Step two: Original studies during the pandemic**

We included original studies that must have been written in English and published in peer-reviewed journals. These studies should be diagnostic accuracy studies. Diagnostic accuracy studies compared results from screening tools (which needed to be validated) with reference standards (the gold standard for determining the presence or absence of disease), validated by measures such as calculated sensitivity and specificity [23–27, 39]. We referred to the guidelines and one BMJ publication by Levis et al. and selected the following reference standards: Diagnostic and Statistical Manual of Mental Disorders (DSM), International Classification of Diseases-10 (ICD-10) criteria, semi-structured interviews, fully structured interviews, and Mini International Neuropsychiatric Interviews (MINI) [6, 10]. According to the results of step one, the screening tools we studied were: PHQ-9, HADS, BDI, CESD, EPDS, and GDS. No restrictions were placed on participants’ age, sex/gender, race, or ethnicity other than that recruitment could not be from psychiatric hospitals, given that the screening was intended to identify undiagnosed depression [9].

We searched the PsycINFO, EMBASE, and MEDLINE databases from March 11, 2020, to April 19, 2022, considering that the World Health Organization defined the COVID-19 pandemic as beginning on March 11, 2020. More than 50% of the recruitment period must have occurred after March 11, 2020, if participant recruitment started before that date. We updated the search on September 7, 2022. The search terms used were adapted from a previous relevant review [10], and the search strategy was developed with the help of the academic librarian (JHS). Details are in Supplementary C.

Two investigators (HQY and JRZ) independently identified potential original studies through title and abstract searches and then independently conducted the full-text review. We emailed the corresponding author to provide us with the full-text article if it was unavailable in the database. All disagreements were resolved by discussion between the two investigators or by consulting the third investigator (LXC).

- **Step three: Meta-analyses before the pandemic**

As four screening tools (i.e., BDI-II, HADS-D, PHQ-9, and GDS-4) were identified from step two, we screened all meta-analysis articles related to the four screening tools from all meta-analysis articles retrieved in step one.

We included meta-analyses that used corresponding versions of the screening tools (some tools have different versions which might influence their optimal cut-offs, for example, GDS [15, 40]), corresponding test populations (e.g., examining optimal cut-offs for the same population as the comparison pandemic study), and corresponding reference standard to each screening tool from original studies during the pandemic.

As in step two, two investigators (JRZ and ZYC) independently identified potential meta-analyses through title and abstract searches and then independently conducted the full-text review.

### Data extraction

Two investigators (HQY and JRZ) extracted data independently, and disagreements were resolved by discussion. Any ambiguity encountered during data extraction was resolved by contacting the corresponding author. We extracted participants’ characteristics (mean age, sex as the female percentage, target population, setting), study design characteristics (country, study design, sample size, number of people with major depression, date of recruitment, screening tools used, reference standards), and publication characteristics (funding source). Based on the DSM or ICD-10, we defined depression as a depressive disorder or a depressive episode. Depressive episodes were prioritized when both (the disorder and episodes) were reported since the screening was intended to detect depressive episodes and further diagnose depressive disorder [11]. We extracted the optimal cut-offs, sensitivity, and specificity from the included original studies.

According to the diagnostic interviews corresponding to each screening tool from original studies during the pandemic, we also extracted the sample number, number of cases, sensitivity, specificity, and optimal cut-off corresponding to the same diagnostic interviews from meta-analyses before the pandemic.

### Risk of bias assessment

Two independent investigators (JRZ and ZYC) assessed the risk of bias in original studies using the Quality Assessment of Diagnostic Accuracy Studies-2 tool (QUADAS-2) [41]. This tool has four domains: patient selection, index test, reference standard, and flow and timing. The first three domains are also assessed in terms of applicability, i.e., the extent to which primary studies apply to the review’s research question [41]. Any disagreements were resolved by consensus or in consultation with the third investigator (LXC). Details are in Supplementary D.

We did not assess the risk of bias for the included meta-analyses because there was currently no suitable tool for systematic reviews of diagnostic accuracy.

### Data analysis

We did not conduct a meta-analysis as 1) PHQ-9, BDI-II, and GDS-4 had only one included original study; 2) HADS-D had two included original studies, but one did not provide the data we needed [42]. We compared changes in the optimal cut-off of the depression tools from included original studies (during the pandemic) with the optimal cut-off of the same tools before the pandemic, as reported in meta-analyses. In one original study, the authors did not report the optimal cut-off. However, the corresponding author provided the raw data, and we calculated the optimal cut-off [43]. The optimal cut-off was the cut-off maximizing Youden’s J statistic (sensitivity + specificity – 1) [12, 43–45]. We also calculated the prevalence of depression in both the included original studies and the meta-analyses before the pandemic. Prevalence was the ratio of the number of people with depression identified by diagnostic interviews within the total number of people who underwent two stages of screening tools and diagnostic interviews [46].

## Results

### Search results

**Fig. 2:**
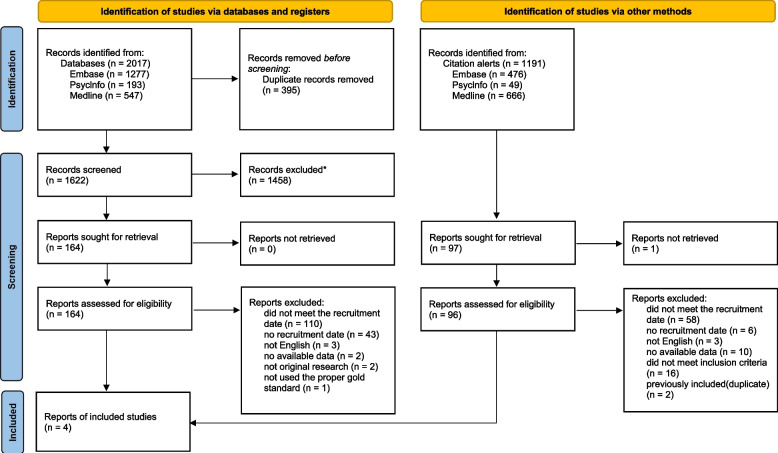
PRISMA flow chart

Figure 2 shows the flowchart of the selection and inclusion processes for original studies. We found 2017 potentially eligible studies identified by database search. After duplicate removal, 1622 studies were screened for titles and abstracts. We reviewed 164 full-text documents. 161 studies were excluded for not meeting the recruitment date (*n* = 110), not reporting the recruitment date (*n* = 43), not having available data (*n* = 2), not being written in English (*n* = 3), or not being classified as original research (*n* = 2), or not using the proper gold standard (*n* = 1). Therefore, we included three eligible studies from our original search. Another record was identified from citation alerts and added. In total, four original studies were included in this review [43, 45, 47, 48].

We found 70 potentially eligible studies identified by database search. After screening for titles and abstracts, 44 meta-analyses were reviewed for full-text: 5 meta-analyses about BDI; 12 meta-analyses about HADS; 22 meta-analyses about PHQ-9; 7 meta-analyses about GDS-4; two of them were meta-analyses of both PHQ-9 and HADS. 40 meta-analyses were excluded based on the version of the screening tools, the test populations, and the reference standards used in the original studies. Finally, we selected the most recent meta-analyses which had the same version of the screening tool and the reference standard when compared with corresponding original studies.

### Characteristics of studies

Table 1 contains the characteristics of the included original studies. Briefly, three out of four studies were published in 2022 [43, 47, 48], one in 2021 [45], and only one was reported to be funded by the National Research Council41. All four studies were cross-sectional, with three studies conducted in Europe [43, 47, 48] and one in Asia [45]. Participant recruitment in two studies began after March 2020 [43, 45] (August 2020 to November 2020, May 2020 to June 2021 respectively), and in the other two before March 2020 but more than half of the entire recruitment period took place after March 2020 [47, 48] (November 2019 to May 2021 and September 2019 to February 2021 respectively). The studies used different screening tools, although the HADS-D (reported independently from HADS-Anxiety) was reported in two studies. Tamrchi et al. used BDI-II and HADS-D, Dering et al. used HADS-D, Pranckeviciene et al. used PHQ-9, and Abdullah et al. used GDS-4 (the version of this subscale was Van Marjwik) [40]. Concerning patient characteristics, three out of four studies reported mean age (22.7y, 44.6y, and 69.0y, respectively) and the ratio of females (51.0, 52.9, and 82.0%, respectively) [43, 45, 47]. Three studies were conducted in outpatient setting [45, 47, 48], and one was conducted in the community [43].

**Table 1:** Characteristics of included original studies

Study ID	Tamrchi, 2021	Dering, 2022	Pranckeviciene, 2022	Abdullah, 2022
**Publication characteristics**				
Funding source	None	None	LMT LT ^a^	None
**Study design characteristics**				
Country	Iran	Germany	Lithuania	United Kingdom
Study design	Cross-sectional	Cross-sectional	Cross-sectional	Cross-sectional
Sample size (number of patients with major depression)	244 (58)	107 (7)	560 (81)	62 (18)
Date of recruitment (YYYY.MM.DD)	2020.8.1 to 2020.11.25	2019.11 to 2021.5	2020.5 to 2021.6	2019.9.1 to 2021.2.28
Screening tool used	HADS-D BDI-II	HADS-D	PHQ-9	GDS-4
Reference standard	MINI	SCID	CIS-R	DSM-5
**Patient characteristics**				
Age (years), mean	44.6	69.0	22.7	–
Sex, Female (%)	129 (52.9%)	55 (51.0%)	459 (82.0%)	–
Target population	Type 2 Diabetes	Chronic Thromboembolic Pulmonary Hypertension	University students	Young adults seen in a memory clinic setting (< 65 years)
Setting	Outpatient	Outpatient	Community	Outpatient

*HADS-D* Hospital Anxiety and Depression Scale-Depression, *BDI-II* Beck Depression Inventory, *PHQ-9* Patient Health Questionnaire-9, *GDS-4* Geriatric Depression Scale-4, *MINI* Mini International Neuropsychiatric Interview, *SCID* Structured Clinical Interview for Diagnostic and Statistical Manual of Mental Disorders, *CIS-R* Clinical Interview Schedule-Revised, *DSM-5* Diagnostic and Statistical Manual of Mental Disorders-5

-Not reported

^a^LMT LT (in Lithuanian - Lietuvos Mokslo Taryba) Research Council of Lithuania

Two meta-analyses were published in 2021 [11, 15], and two in 2019 [10, 12]. Von Glischinski et al. included 27 studies with 11,026 participants about BDI-II; Wu et al. included 101 studies with 22,574 participants about HADS-D; Levis et al. included 58 studies with 17,357 participants about PHQ-9; Branez-Condorena et al. included 7 studies with 1774 participants about GDS-4. Besides, considering test populations, we couldn’t find any suitable meta-analyses.

### Risk of bias

**Fig. 3:**
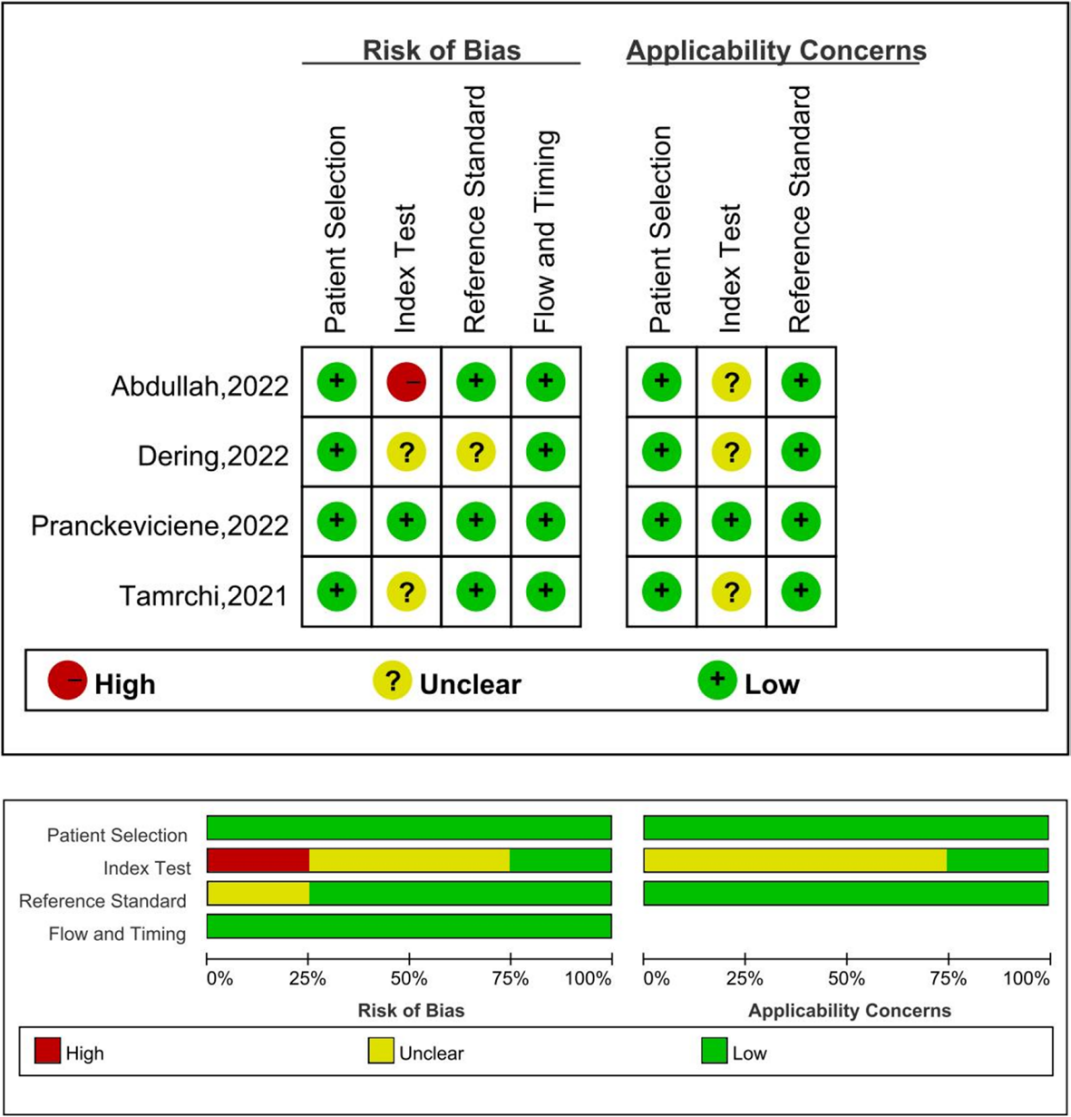
Risk of bias of each original study

Figure 3 shows the risk of bias assessment for each original study. The study by Tamrchi et al. was judged as having some concerns in domain 2: index test and a low risk of bias in the other domains. Concerning applicability, it was judged as having some concern in domain 2: index tests and a low risk of bias in the other domains [45]. The study by Pranckeviciene et al. was judged as having a low risk of bias in all domaints [43]. The study by Dering et al. was judged as having some concerns in domain 2: index test and domain 3: reference standards and having a low risk of bias in the other domains. Concerning applicability, it was judged as having some concern in domain 2: index test and a low risk of bias in the other domains [47]. The study by Abdullah et al. was judged as having a high risk of bias in domain 2: index test and a low risk of bias in the other domains. Concerning applicability, it was judged as having some concern in domain 2: index test and a low risk of bias in the other domains [48]. Overall, the index test was the driver of the most concerns concerning the risk of bias. Details are in Supplementary F.

Table 2 shows comparison of before and during the pandemic depression screening tools diagnosis accuracy.

**Table 2:** Comparison of before and during the pandemic depression screening tools diagnosis accuracy

**During the pandemic**							
Study ID	Tool name	Sensitivity	Specificity	Optimal cut-off	Prevalence (%)	Reference standard	Target population
Tamrchi, 2021 [45]	BDI-II	0.84	0.99	14	23.8	MINI	Type 2 Diabetes
	HADS-D	0.97	0.95	10	23.8	MINI	
Dering, 2022 [47]	HADS-D	0.57	0.92	11 ^a^	6.5	SCID	Chronic Thromboembolic Pulmonary Hypertension
Pranckeviciene, 2022 [43]	PHQ-9	0.78	0.67	11	14.5	CIS-R	University students
Abdullah, 2022 [48]	GDS-4	0.79	0.23	1.75	29.0	DMS-5	Young adults seen in a memory clinic setting (< 65 years)
**Before the pandemic**							
Study ID	Tool name	Sensitivity	Specificity	Optimal cut-off	Prevalence (%)	Reference standard	Target population
von Glischinski, 2018 [12]	BDI-II	0.86	0.78	14.5	17.6	–	Psychiatric patients, primary care, healthy populations
Wu, 2021 [11]	HADS-D	0.75	0.80	7	15.0	MINI	Medically ill patients ^b^
		0.82	0.78	7	9.8	SCI	
Levis, 2019 [10]	PHQ-9	0.82	0.75	8	10.9	FCI	Unrestricted population ^c^
Brañez-Condorena, 2021 [15]	GDS-4	0.85	0.67	3	18.5	DMS	Older adults

*HADS-D* Hospital Anxiety and Depression Scale-Depression, *BDI-II* Beck Depression Inventory, *PHQ-9* Patient Health Questionnaire-9, *GDS-4* Geriatric Depression Scale-4

*MINI* Mini International Neuropsychiatric Interview, *SCID* Structured Clinical Interview for Diagnostic and Statistical Manual of Mental Disorders, *CIS-R* Clinical Interview Schedule-Revised, *DSM-5* Diagnostic and Statistical Manual of Mental Disorders-5, *SCI* Semi Structured Interview, *FCI* Fully Structured Interview

-No reference standard

^a^Not Optimal Cut-off

^b^Chronic physical health problems, such as patients with cancer, chronic heart disease, and chronic obstructive pulmonary disease

^c^Patients with cancer, patients with chronic diseases, elderly population, health population, etc

### BDI-ii (0–63)

The study by Tamrchi et al. reported that using the MINI as the reference standard, the optimal cut-off for BDI-II when screening patients with type 2 diabetes (244 participants, mean age 44.6 years, female ratio 52.9%) for major depression was 14, with sensitivity and specificity of 0.84 and 0.99. The prevalence of major depression in their study was 23.8% [45].

A meta-analysis by von Glischinski et al. based on studies before the pandemic (27 included studies with 11,026 participants) reported that the optimal cut-off for BDI-II when screening psychiatric, primary care, and healthy populations for major depression was 14.5, with sensitivity and specificity of 0.86 and 0.78. Notably, they did not report the reference standards used. The prevalence of major depression in this pre-pandemic meta-analysis was 17.6% [12].

### HADS-d (0–21)

The study by Tamrchi et al. reported that using MINI as the reference standard, the optimal cut-off for HADS-D when screening patients with type 2 diabetes (with 244 participants, mean age 44.6, female ratio 52.9%) for major depression was 10, with sensitivity and specificity of 0.97 and 0.95. The prevalence of major depression in their study was 23.8% [45]. The study by Dering et al. reported that using the Structured Clinical Interview for the Diagnostic and Statistical Manual of Mental Disorders (SCID: semi-structured interview) as the reference standard, the cut-off of HADS-D in screening patients with chronic thromboembolic pulmonary hypertension (107 participants, mean age 69.0 years, female ratio 51%) for major depression was 11, with sensitivity and specificity of 0.57 and 0.92. The prevalence of major depression in their study was 6.5%. However, this study only provided the ROC curve of HADS-D and the sensitivity and specificity when the cut-off was 11, with no additional data to indicate the optimal cut-off [47].

A meta-analysis by Wu et al. based on studies before the pandemic (101 studies with 22,574 participants) reported that the optimal cut-off for HADS-D when screening medically ill patients (chronic physical health problems, such as patients with cancer, chronic heart disease, and chronic obstructive pulmonary disease) for major depression was 7, with sensitivity and specificity of 0.82 and 0.78, 0.81 and 0.73, and 0.75 and 0.80 when referenced to semi-structured interviews, fully structured interviews, or MINI, respectively. The corresponding prevalence of major depression in this pre-pandemic meta-analysis was 9.8, 8.7, and 15.0%. The accuracy of all cut-offs was similar across reference standards, subgroups, and studies that did not report results (no statistical difference). Choosing a higher cut-off for identifying medically ill patients reduced false positives [11].

### PHQ-9 (0–27)

The study by Pranckeviciene et al. reported that using Clinical Interview Schedule-Revised (CIS-R: fully structured interview) as the reference standard, the optimal cut-off for PHQ-9 when screening university students (560 participants, mean age 22.7 years, female ratio 82.0%) for depressive episodes was 11, with sensitivity and specificity of 0.78 and 0.67. The prevalence of depressive episodes in their study was 14.5%. We calculated the sensitivity and specificity of each cut-off according to the original data provided by the authors and selected the corresponding optimal cut-off according to the definition [43]. Details are in Supplementary E.

A meta-analysis by Levis et al. based on studies before the pandemic (58 studies with 17,357 participants) reported that using fully structured interviews as the reference standard, the optimal cut-off for PHQ-9 when screening the unrestricted population (Patients with cancer, patients with chronic diseases, elderly population, health population, etc.) for major depression was 8, with sensitivity and specificity of 0.82 and 0.75. The prevalence of major depression in this pre-pandemic meta-analysis was 10.9%. This prevalence was the ratio of the number of people diagnosed with major depression through fully structured interviews among the total number of people who received PHQ-9 and fully structured interviews at both stages. In this meta-analysis, 14 studies used fully structured interviews as the reference standard, with a total number of 7680 people, among which 839 had major depression [10].

### GDS-4 (0–4)

The study by Abdullah et al. reported that using the DSM-5 as the reference standard, the optimal cut-off when screening adults seen in a memory clinic setting (< 65 years) (62 participants, without reported mean age and sex ratio) for depression was 1.75, with sensitivity and specificity of 0.79 and 0.23. This low specificity was based on the study’s limitation, as it did not routinely screen for GDS-4 in patients who were not clinically suspected of depression. The prevalence of depression in this study was 29.0% [48].

A meta-analysis by Branez-Condorena et al. based on studies before the pandemic reported that using DMS or ICD-10 as the reference standard, the optimal cut-off when screening older adults for depression was 3, with sensitivity and specificity of 0.85 and 0.67. The prevalence of depression in this pre-pandemic meta-analysis was 18.5%. In this meta-analysis, we extracted the GDS-4 studies of the same version as the included study. A total of 7 studies included 1774 people, 328 of whom had depression [15].

## Discussion

### Main findings

In this systematic review, based on four original studies with four depression screening tools, we found that optimal cut-offs of HADS-D, PHQ-9, and GDS-4 may have changed, while we had no evidence that the optimal cut-off of BDI-II had changed. We also found that the prevalence of depression in these studies during the pandemic was higher than in meta-analyses of depression cut-offs before the pandemic, although this prevalence difference might be at least partly due to differences in the tested populations: Studies conducted during the pandemic the recruited participants from specific target populations (two studies included people with type 2 diabetes and chronic thromboembolic pulmonary hypertension, one included adults at a memory clinic, and one included University students). We compared optimal depression cut-offs in these studies conducted during the pandemic with optimal cut-offs identified in meta-analyses. In terms of risk of bias, three out of four included original studies were rated as having some concerns, mainly because it was unclear whether screening thresholds had been specified in advance.

### Comparison with previous studies

To our knowledge, this is the first study to consider spectrum effects in commonly used measures of depression and compare the changes in optimal cut-offs of the depression screening tools since the pandemic. Although indicative, the findings of this study show optimal cut-offs of several screening tools (e.g., PHQ-9 and HADS-D) that might be sensitive to changes in different study populations and reference standards.

### Implications

We tried to retrieve 24 screening tools according to guidelines [1, 4–6] (details are in Supplementary E), but we found only four screening tools were studied during the pandemic. This suggested that future studies of diagnostic cut-offs should pay more attention to other screening tools (e.g., CESD and EPDS).

The guidelines recommended screening in primary care settings [1, 4], but we found that most of the screenings studied were performed in secondary care settings. Another study also showed that 79% of antidepressant prescriptions were written by non-mental health care providers [49, 50], so future research should also focus more on screening for depression in the primary care population. In addition, patients with specific chronic diseases (e.g., Chronic gastrointestinal disease) in secondary-care settings could have decreased appetite, weight loss, or insomnia, which also showed up on screening questionnaires for depression [25]. If we use the tools recommended by the guidelines for screening in primary care settings and their corresponding cut-offs, we may identify more patients with depression among these populations and then give them antidepressants. This action might bring more harm than benefit as antidepressants might not work and bring some adverse effects (e.g., hypertension and impaired sexual function) [4]. Changing cut-offs for screening tools used in primary care settings or developing and using population-specific screening tools might help prevent unnecessary treatments.

Our original goal was to determine whether the COVID-19 pandemic would affect screening tools’ cut-offs. Our study found that optimal cut-offs of HADS-D, PHQ-9, and GDS-4 may be changed, while the cut-off of BDI-II was less likely to change. However, Levis, B. et al. found that the diagnostic accuracy of HADS-D did not differ across reference standards or participant characteristics [11], and the diagnostic accuracy of PHQ-9 under the semi-structured reference standard was higher than other reference standards and the specificity was higher in the elderly [10]. There were no pre-pandemic meta-analyses showing the diagnostic accuracy of GDS-4 and BDI under different study populations and reference standards had changed. Based on the aforementioned, our study indicated that the optimal cut-off for different screening tools may be sensitive to changes in study populations and reference standards. Further confirmatory studies are needed in the future. This also suggested that future studies should also pay more attention to the changes in diagnostic accuracy of other screening tools when using different reference standards and studying different populations. In addition, we found that the proposed screening tools do not have official cut-offs for different age groups. This suggest that we should consider cut-offs for different age groups, as instruments seem to have a spectrum effect in future research avenues.

### Limitations

First, one of the included original studies had a high risk of bias, however, it was the only study about GDS-4. Thus, we could not exclude it from performing a sensitivity analysis, which means we should be cautious in interpreting the results of GDS-4. Second, we only focused on the English language screening tool, so we must exercise caution when generalizing results to other language versions of the screening tool. Third, although we did not restrict geography when searching for guides, meta-analyses, or original studies, three of the four included studies were from Europe. Our results were based on data from only a limited number of countries. Thus, we should exercise caution when generalizing result across the globe. Finally, the difference in the study population, the type of reference standard used, and the result of the meta-analysis compared with a single study prevents us from concluding that the change in optimal cut-offs was due to the pandemic. However, the prevalence can change due to different circumstances and these findings have wider implications and open an important avenue for future research.

## Conclusion

In this review, we found that the optimal cut-off for different screening tools may be sensitive to changes in study populations and reference standards. In addition, we identified potential spectrum effects that should be considered in post-COVID time, aiming to improve diagnostic accuracy, by investigating and possibly establishing cut-offs in different populations when prevalence is proved to be different, i.e., in adolescents, young, middle-aged and older adults. Given the small number of included original studies and lack of evidence for other available tools (e.g., CES-D and EPDS), further validation studies are still required.

### Supplementary Information

**Additional file 1.**

## Data Availability

All data generated or analyzed during this study are included in this published article [and its supplementary information files].

## Abbreviations

USPSTF: United states preventive services task force
NICE: United kingdom national institute for health and care excellence
PHQ-9: Patient health questionnaire-9
HADS: Hospital anxiety and depression scale
BDI: Beck depression inventory
CES-D: Center for epidemiologic studies depression
EPDS: Edinburgh postnatal depression scale
GDS: Geriatric depression scale
HAM-D: Hamilton depression rating scale
PDSS: Postpartum depression screening scale
CDS: Cardiac depression scale
MFQ-SF: Moods and feelings questionnaire, short form
EQ-5D: EuroQol five dimensions questionnaire
MADRS: Montgomery-åsberg depression rating scale
SPSI-RTM: Social problem-solving inventory-revised
BASC: Behavior assessment system for children
CBCL: Child behavior checklist
CDI: Children’s depression inventory
CDRS: Children’s depression rating scale
BHS: Beck hopelessness scale
QIDS-SR: Quick inventory of depressive symptomatology-self-report
RFS: Reminiscence functions scale
SF-36: Short form health survey
SAS-SR™: Social adjustment scale-self report
SFQ: Social functioning questionnaire
PRISMA-DTA: Preferred reporting items for systematic review and meta-analysis of diagnostic test accuracy studies
APA: American psychological association
RANZCP: the Royal australian and new zealand college of psychiatrists clinical practice
DSM: Diagnostic and statistical manual of mental disorders
ICD-10: International classification of diseases-10 criteria
MINI: Mini international neuropsychiatric interviews
QUADAS-2: the Quality assessment of diagnostic accuracy studies-2 tool
SCID: Structured clinical interview for the diagnostic and statistical manual of mental disorders
CIS-R: Clinical interview schedule-revised
