# Supplementary material for: Optimal cut-offs of depression screening tools during the COVID-19 pandemic: a systematic review
Source: BMC Psychiatry. 2023 Dec 19;23:953. doi: 10.1186/s12888-023-05455-8 (PMC10729515; doi:10.1186/s12888-023-05455-8)
Supplement: Supplementary file 1 — Additional file 1. [file 12888_2023_5455_MOESM1_ESM.docx]

**Supplement Material**

**Item**

**Supplementary A.** Search strategies for depression screening guidelines

**Supplementary B.** Screening tool names

**Supplementary C.** Search strategies for original studies

**Supplementary D.** QUADAS-2 coding manual for original studies included in the present study

**Supplementary E.** The processing of raw data

**Supplementary F.** QUADAS-2 ratings for each included original study

**Supplementary A.** Search strategies for depression screening guidelines

We searched the PsycINFO, EMBASE, and MEDLINE databases from January 1, 2012, to January 1, 2022 and restricted the language to English. The search terms were as follows.

Two investigators (JRZ and ZYC) independently identified potential studies through title and abstract searches and then independently conducted the full-text review. All disagreements were resolved by discussion between the two investigators or by consulting the third investigator (LXC).

We found 5,193 potentially eligible studies identified by database search. After duplicate removal, 4,934 studies were screened for titles and abstracts. We reviewed 94 full-text documents. 73 studies were excluded. Therefore, we included 21eligible studies. There are the Postpartum Depression Screening Scale (PDSS) and short-form Cardiac Depression Scale (CDS), which have not been covered by previous work. A supplementary search was conducted on meta-analysis studies related to PDSS and CDS, but no relevant studies were found.


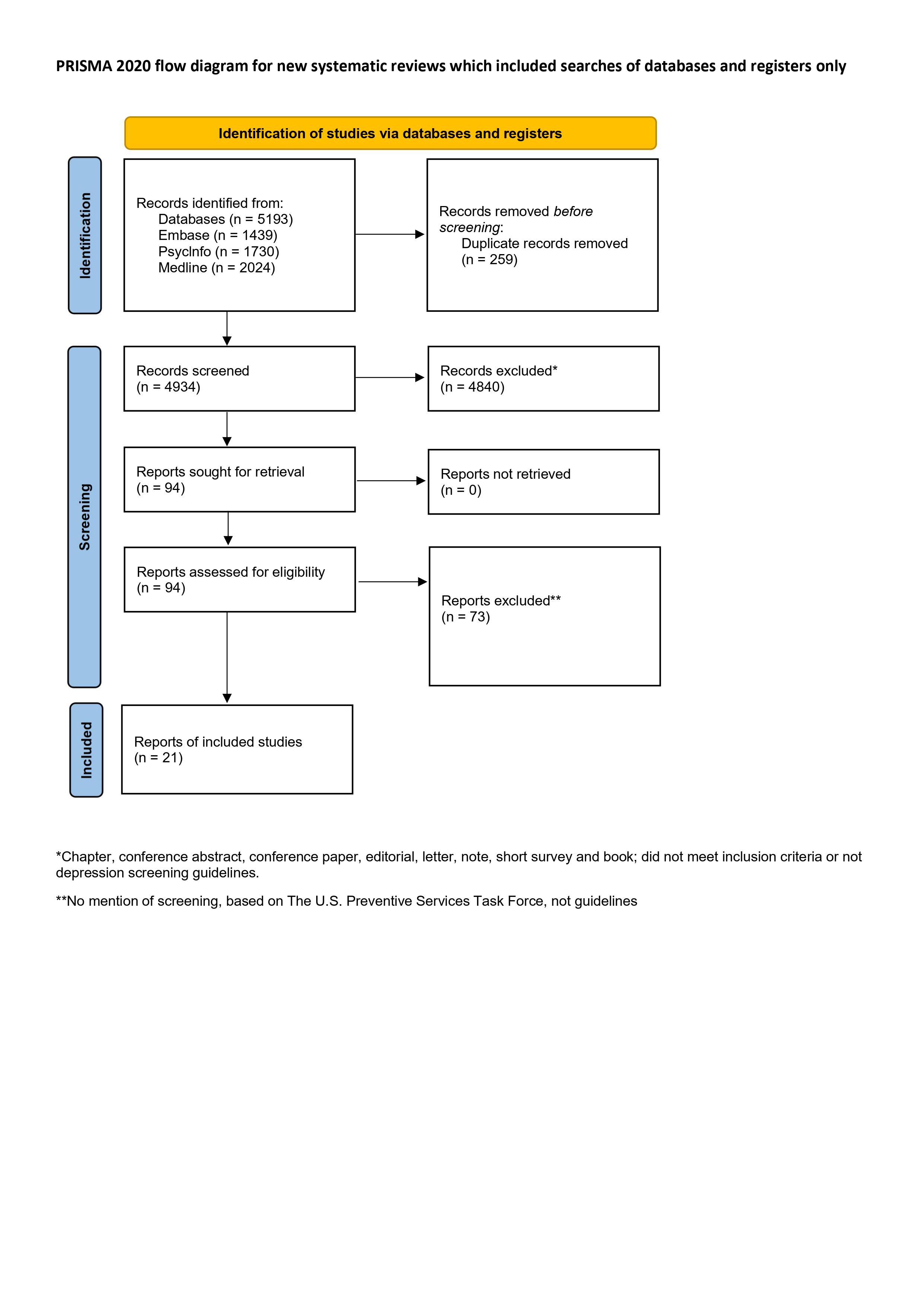


**Databse: MEDLINE**1 Screening/
2 Health Screening/
3 Screening Tests/
4 screen*.af.
5 1 or 2 or 3 or 4
6 "guideline*".ti.
7 "guidance*".ti.
8 position paper.ti.
9 position stand.ti.
10 statement.ti.
11 recommendation.ti.
12 consensus.ti.
13 practice parameter.ti.
14 standards.ti.
15 management.ti.
16 6 or 7 or 8 or 9 or 10 or 11 or 12 or 13 or 14 or 15
17 Mental Disorders/
18 Mood Disorders/
19 depression.af.
20 "depress*".af.
21 17 or 18 or 19 or 20
22 5 and 16 and 21
23 limit 22 to (english language and yr="2012 - 2022")

**Database:EMBASE**

1.'depression'/exp

2.'mental disease'/exp

3.'mood disorder'/exp

4.'major depression'/exp

5.'depressive disorder':ab,ti

6.1 or 2 or 3 or 4 or 5

7.guideline*ti

8.guidance*.ti

9.'position paper'ti

10.'position stand'ti

11.statement*.ti

12.recommendation*.ti

13.consensus.:ti

14.practice parameter* ti

15.standardst

16.management*.ti

17.7 or 8 or 9 or 10 or 11 or 12 or 13 or 14 or 15 or 16

18.'screening/exp

19.'mass screeningexp

20.screen*.ab,ti

21.18 or 19 or 20

22.6 and 17 and 21

23. 22 and (englishj/lim and (2012-2022]/py

**Databse: APA PsycInfo <1806 to September Week 3 2023>**1 Screening/
2 Health Screening/
3 Screening Tests/
4 screen*.af.
5 1 or 2 or 3 or 4
6 "guideline*".ti.
7 "guidance*".ti.
8 position paper.ti.
9 position stand.ti.
10 statement.ti.
11 recommendation.ti.
12 consensus.ti.
13 practice parameter.ti.
14 standards.ti.
15 management.ti.
16 6 or 7 or 8 or 9 or 10 or 11 or 12 or 13 or 14 or 15
17 Mental Disorders/
18 Mood Disorders/
19 depression.af.
20 "depress*".af.
21 17 or 18 or 19 or 20
22 5 and 16 and 21
23 limit 22 to (english language and yr="2012 - 2022")

**Supplementary B. Screening tool names**

| **Guideline** | **Population** | **Screening tools** |
| --- | --- | --- |
| US Preventive Services Task Force(USPSTF)^1-10^ | Adolescents aged 12 to 18 years | Patient Health Questionnaire (PHQ-9) |
|  |  | PHQ modified for adolescents (PHQ-A) |
|  |  | Center for Epidemiologic Studies Depression Scale(CESD) |
|  | Adults, including pregnant and postpartum persons, and older adults (65 years or older) | Patient Health Questionnaire (PHQ) |
|  |  | Center for Epidemiologic Studies Depression Scale (CESD) |
|  |  | Geriatric Depression Scale (GDS) |
|  |  | Edinburgh Postnatal Depression Scale (EPDS) |
| UK National Institute for Health and Clinical Excellence(NICE)^11-13^ | People aged 18 and over | Patient Health Questionnaire (PHQ-9) |
|  | Children and young people aged 5 to 18 years | Self‑report questionnaire for depression(NA)* |
|  | People aged 18 and over who also have a chronic physical health problem such as cancer, heart disease or diabetes. | Whooley questions† |
|  | Perinatal women | Whooley questions† plus help question‡ |
| American College of Obstetricians and Gynecologists(ACOG)^14^ | Women during pregnancy | Edinburgh Postnatal Depression Scale (EPDS) |
|  |  | Patient Health Questionnaire-9 (PHQ-9) |
|  |  | Beck Depression Inventory (BDI) |
|  |  | Center for Epidemiologic Studies Depression Scale (CESD) |
| Australian Clinical Practice Guidelines(ACPG)^15,16^ | Perinatal women | Edinburgh Postnatal Depression Scale (EPDS) |
|  |  | Patient Health Questionnaire (PHQ-9) |
| American Society of Clinical Oncology Guideline Adaptation(ASCO)^17^ | Adults (age 18 years and older) at any phase of the cancer continuum and regardless ofcancer type, disease stage, or treatment modality. | Patient Health Questionnaire (PHQ-9) |
| The 2020 Royal Australian and New Zealand College of Psychiatrists clinical practice guidelines(MDcpg^2020^) ^18^ | All peoeple | DSM-5(diagnosis)** |
|  |  | ICD-11(diagnosis)** |
| 2022 U.S. Department of Veterans Affairs and U.S. Department of Defense Clinical Practice Guideline^19^ | All people | NA* |
| American Academy of Pediatrics(AAP)^8^ | Perinatal women | Edinburgh Postnatal Depression Scale (EPDS) |
|  |  | Patient Health Questionnaire-9 (PHQ-9) |
|  |  | Patient Health Questionnaire-2 (PHQ-2) |
|  |  | Beck Depression Inventory (BDI-II) |
|  |  | Hamilton Depression Rating Scale (HAM-D) |
|  |  | Postpartum Depression Screening Scale (PDSS) |
| American College of Physicians(ACP)^6^ | Youth aged 10 to 21 years | Universal adolescent depression self-report instruments(NA)* |
| American Academy of Family Physicians (AAFP)^20^ | Depression After ACS Events | Beck Depression Inventory (BDI-II) |
|  |  | Geriatric Depression Scale (GDS) |
|  |  | Patient Health Questionnaire-9 (PHQ-9) |
|  |  | Hospital and Anxiety Depression Scale(HADS) |
| International Committee on Mental Health in CF (ICMH)^21^ | Adolescents and adults with CF (ages 12– adulthood) | Patient Health Questionnaire-9 (PHQ-9) |
|  | Children and adolescents with CF (ages 0–17) | Patient Health Questionnaire-9 (PHQ-9) |
|  |  | Patient Health Questionnaire-8 (PHQ-8) |
|  |  | Patient Health Questionnaire-2 (PHQ-2) |
| National Heart Foundation of Australia (NHFA)^22^ | Depression in patients with recurrent coronary heart disease (CHD) | Patient Health Questionnaire-2 (PHQ-2) |
|  |  | Short-form Cardiac Depression Scale (CDS) |
| The North American Menopause Society (NAMS) and the Women and Mood Disorders Task Force of the National Network of Depression Centers^23^ | Perimenopausal | Patient Health Questionnaire (PHQ-9) |
| Clinical guidelines for depression screening in youth diagnosed with IBD^24^ | Adolescents with IBD ages 12 | Moods and Feelings Questionnaire, Short Form (MFQ-SF) |
|  | Adolescents with IBD ages 13 | Patient Health Questionnaire-9 (PHQ-9) |
| American Psychological Association(APA)^25^ | Across the Lifespan | Beck Depression Inventory (BDI) |
|  |  | Center for Epidemiologic Studies Depression Scale (CESD) |
|  |  | EuroQol Five Dimensions Questionnaire (EQ-5D) |
|  |  | Hamilton Depression Rating Scale (HAM-D) |
|  |  | Montgomery-Åsberg Depression Rating Scale (MADRS) |
|  |  | Social Problem-Solving Inventory-Revised (SPSI-RTM) |
|  | Children and Adolescents | Behavior Assessment System for Children (BASC) |
|  |  | Child Behavior Checklist (CBCL) |
|  |  | Children’s Depression Inventory (CDI) |
|  |  | Children’s Depression Rating Scale (CDRS) |
|  | General Adult Population | Beck Hopelessness Scale (BHS) |
|  |  | Quick Inventory of Depressive Symptomatology-Self-Report (QIDS-SR) |
|  |  | Patient Health Questionnaire (PHQ-9) |
|  |  | Reminiscence Functions Scale (RFS) |
|  |  | Short Form Health Survey (SF-36) |
|  |  | Social Adjustment Scale-Self Report (SAS-SR)^TM^ |
|  |  | Social Functioning Questionnaire (SFQ) |
|  | Older Adults | Geriatric Depression Scale (GDS) |
|  |  | Life Satisfaction Index |

*:No specific screening name

**: Diagnosis standard, not screening tool

Whooley questions† :During the last month, have you often been bothered by feeling down, depressed or hopeless? During the last month, have you often been bothered by having little interest or pleasure in doing things?

plus help question‡:If the patient answers Yes to either of the Whooley questions, a third question should be considered: Is this something you feel you need or want help with?

**Supplementary C. Search strategies**

**Database: Ovid MEDLINE(R) ALL <1946 to September 06, 2022>**

1. Mass Screening/
2. Psychiatric Status Rating Scales/
3. Primary Care Evaluation of Mental Disorders/
4. "Predictive Value of Tests"/
5. "Reproducibility of Results"/
6. exp "Sensitivity and Specificity"/
7. Psychometrics/
8. Prevalence/
9. Reference Values/
10. Reference Standards/
11. exp Diagnostic Errors/
12. validation studies.pt.
13. comparative study.pt.
14. screen*.af.
15. prevalence.af.
16. detect*.ti.
17. recogni*.ti.
18. sensitiv*.ti.
19. valid*.ti.
20. revalid*.ti.
21. predict*.ti.
22. accura*.ti.
23. psychometric*.ti.
24. identif*.ti.
25. specificit*.ab.
26. cut?off*.ab.
27. cut* score*.ab.
28. cut?point*.ab.
29. threshold score*.ab.
30. reference standard*.ab.
31. reference test*.ab.
32. index test*.ab.
33. gold standard.ab.
34. reliab*.ab.
35. 1 or 2 or 3 or 4 or 5 or 6 or 7 or 8 or 9 or 10 or 11 or 12 or 13 or 14 or 15 or 16 or 17 or 18 or 19 or 20 or 21 or 22 or 23 or 24 or 25 or 26 or 27 or 28 or 29 or 30 or 31 or 32 or 33 or 34
36. Mental disorders/di, pc
37. Mood disorders/di, pc
38. depressive disorder/di, pc
39. depression, postpartum/di, pc
40. depression/di, pc
41. dysthym*.tw,kf.
42. blues.tw,kf.
43. melanchol*.tw,kf.
44. 36 or 37 or 38 or 39 or 40 or 41 or 42 or 43
45. "Hospital Anxiety and Depression Scale Depression scale".af.
46. HADS.af.
47. 45 or 46
48. 35 and 44 and 47
49. limit 48 to yr="2022 -Current"
50. "Patient Health Questionnaire 9".af.
51. PHQ-9.af.
52. 50 or 51
53. 35 and 44 and 52
54. limit 53 to yr="2022 -Current"
55. "Beck Depression Inventory".af.
56. 35 and 44 and 55
57. limit 56 to yr="2022 -Current"
58. "Center for Epidemiologic Studies Depression Scale".af.
59. CES-D.af.
60. CESD.af.
61. 58 or 59 or 60
62. 35 and 44 and 61
63. limit 62 to yr="2022 -Current"
64. "Geriatric Depression Scale".af.
65. 35 and 44 and 64
66. limit 65 to yr="2022 -Current"
67. 49 or 54 or 57 or 63 or 66

**Database: EMBASE**

1. 'mass screening'/exp
2. 'psychological rating scale'/exp
3. 'primary care evaluation of mental disorders'/exp
4. 'predictive value'/exp
5. 'reproducibility'/exp
6. 'sensitivity and specificity'/exp
7. 'psychometry'/exp
8. 'prevalence'/exp
9. 'reference value'/exp
10. 'standard'/exp
11. 'diagnostic error'/exp
12. 'validation study'/exp
13. 'comparative study'/exp
14. screen*:ti,ab,kw
15. prevalence:ti,ab,kw
16. detect*:ti
17. recogni*:ti
18. sensitiv*:ti
19. valid*:ti
20. revalid*:ti
21. predict*:ti
22. accura*:ti
23. psychometric*:ti
24. identif*:ti
25. specificit*:ab
26. cut?off*:ab
27. cut* AND score*:ab
28. cut?point*:ab
29. threshold AND score*:ab
30. reference AND standard*:ab
31. reference AND test*:ab
32. index AND test*:ab
33. gold AND standard:ab
34. reliab*:ab
35. #1 OR #2 OR #3 OR #4 OR #5 OR #6 OR #7 OR #8 OR #9 OR #10 OR #11 OR #12 OR #13 OR #14 OR #15 OR #16 OR #17 OR #18 OR #19 OR #20 OR #21 OR #22 OR #23 OR #24 OR #25 OR #26 OR #27 OR #28 OR #29 OR #30 OR #31 OR #32 OR #33 OR #34
36. 'depression'/exp/dm_di,dm_pc
37. dysthymia:ti,ab,kw
38. blues:ti,ab,kw
39. melanchol*:ti,ab,kw
40. #36 OR #37 OR #38 OR #39
41. hads*
42. hospital AND anxiety AND depression AND scale
43. #41 OR #42
44. #35 AND #40 AND #43
45. #35 AND #40 AND #43 AND [embase]/lim AND [2022-2022]/py
46. patient AND health AND questionnaire AND 9
47. #35 AND #40 AND #46
48. #35 AND #40 AND #46 AND [embase]/lim AND [2022-2022]/py
49. beck AND depression AND inventory
50. #35 AND #40 AND #49
51. #35 AND #40 AND #49 AND [embase]/lim AND [2022-2022]/py
52. center AND for AND epidemiologic AND studies AND depression AND scale
53. 'ces d'
54. Cesd
55. #52 OR #53 OR #54
56. #35 AND #40 AND #55
57. #35 AND #40 AND #55 AND [embase]/lim AND [2022-2022]/py
58. geriatric AND depression AND scale
59. #35 AND #40 AND #58
60. #35 AND #40 AND #58 AND [embase]/lim AND [2022-2022]/py
61. #45 OR #48 OR #51 OR #57 OR #60

**Database: APA PsycINFO <1806 to August Week 5 2022>**

1. Diagnosis/
2. Medical Diagnosis/
3. Psychodiagnosis/
4. Misdiagnosis/
5. Screening/
6. Health Screening/
7. Screening Tests/
8. Prediction/
9. Cutting Scores/
10. Psychometrics/
11. Test Validity/
12. screen*.af.
13. predictive value*.af.
14. detect*.ti.
15. sensitiv*.ti.
16. valid*.ti.
17. revalid*.ti.
18. accura*.ti.
19. psychometric*.ti.
20. specificit*.ab.
21. cut?off*.ab.
22. cut* score*.ab.
23. cut?point*.ab.
24. threshold score*.ab.
25. reference standard*.ab.
26. reference test*.ab.
27. index test*.ab.
28. gold standard.ab.
29. 1 or 2 or 3 or 4 or 5 or 6 or 7 or 8 or 9 or 10 or 11 or 12 or 13 or 14 or 15 or 16 or 17 or 18 or 19 or 20 or 21 or 22 or 23 or 24 or 25 or 26 or 27 or 28
30. exp "Depression (Emotion)"/
31. dysthym*.tw.
32. blues.tw.
33. melanchol*.tw.
34. 30 or 31 or 32 or 33
35. "Hospital Anxiety and Depression Scale Depression scale".af.
36. HADS.af.
37. 35 or 36
38. 29 and 34 and 37
39. limit 38 to yr="2022 -Current"
40. "Patient Health Questionnaire 9".af.
41. PHQ-9.af.
42. 40 or 41
43. 29 and 34 and 42
44. limit 43 to yr="2022 -Current"
45. "Beck Depression Inventory".af.
46. 29 and 34 and 45
47. limit 46 to yr="2022 -Current"
48. "Center for Epidemiologic Studies Depression Scale".af.
49. CES-D.af.
50. CESD.af.
51. 48 or 49 or 50
52. 29 and 34 and 51
53. limit 52 to yr="2022 -Current"
54. "Geriatric Depression Scale".af.
55. 29 and 34 and 54
56. limit 55 to yr="2022 -Current"
57. 39 or 44 or 47 or 53 or 56

It should be emphasized that the Edinburgh Postnatal Depression Scale (EPDS), a screening tool for depression recommended by US Preventive Services Task Force (USPSTF), was omitted in the formulation of the search strategy. This was our fault, so we retrieved the tool with the following retrieval strategy. We included a total of 684 studies, two investigators (JRZ and ZYC) independently identified potential studies through titles and abstracts, and then independently conducted the full-text review, and disagreements were resolved by discussion, with a third senior investigator (LXC) consulted if necessary. However, we didn't find any that met the criteria.

**Database: Ovid MEDLINE(R) ALL <1946 to February 08, 2023>**

1. Mass Screening/
2. Psychiatric Status Rating Scales/
3. Primary Care Evaluation of Mental Disorders/
4. "Predictive Value of Tests"/
5. "Reproducibility of Results"/
6. exp "Sensitivity and Specificity"/
7. Psychometrics/
8. Prevalence/
9. Reference Values/
10. Reference Standards/
11. exp Diagnostic Errors/
12. validation studies.pt.
13. comparative study.pt.
14. screen*.af.
15. prevalence.af.
16. detect*.ti.
17. recogni*.ti.
18. sensitiv*.ti.
19. valid*.ti.
20. revalid*.ti.
21. predict*.ti.
22. accura*.ti.
23. psychometric*.ti.
24. identif*.ti.
25. specificit*.ab.
26. cut?off*.ab.
27. cut* score*.ab.
28. cut?point*.ab.
29. threshold score*.ab.
30. reference standard*.ab.
31. reference test*.ab.
32. index test*.ab.
33. gold standard.ab.
34. reliab*.ab.
35. 1 or 2 or 3 or 4 or 5 or 6 or 7 or 8 or 9 or 10 or 11 or 12 or 13 or 14 or 15 or 16 or 17 or 18 or 19 or 20 or 21 or 22 or 23 or 24 or 25 or 26 or 27 or 28 or 29 or 30 or 31 or 32 or 33 or 34
36. Mental disorders/di, pc
37. Mood disorders/di, pc
38. depressive disorder/di, pc
39. depression, postpartum/di, pc
40. depression/di, pc
41. dysthym*.tw,kf.
42. blues.tw,kf.
43. melanchol*.tw,kf.
44. 36 or 37 or 38 or 39 or 40 or 41 or 42 or 43
45. EPDS.af.
46. Edinburgh Postnatal Depression.af.
47. Edinburgh Depression Scale.af.
48. 45 or 46 or 47
49. 35 and 44 and 48
50. limit 49 to yr="2020 -Current"

**Database: EMBASE**

1. 'mass screening'/exp
2. 'psychological rating scale'/exp
3. 'primary care evaluation of mental disorders'/exp
4. 'predictive value'/exp
5. 'reproducibility'/exp
6. 'sensitivity and specificity'/exp
7. 'psychometry'/exp
8. 'prevalence'/exp
9. 'reference value'/exp
10. 'standard'/exp
11. 'diagnostic error'/exp
12. 'validation study'/exp
13. 'comparative study'/exp
14. screen*:ti,ab,kw
15. prevalence:ti,ab,kw
16. detect*:ti
17. recogni*:ti
18. sensitiv*:ti
19. valid*:ti
20. revalid*:ti
21. predict*:ti
22. accura*:ti
23. psychometric*:ti
24. identif*:ti
25. specificit*:ab
26. cut?off*:ab
27. cut* AND score*:ab
28. cut?point*:ab
29. threshold AND score*:ab
30. reference AND standard*:ab
31. reference AND test*:ab
32. index AND test*:ab
33. gold AND standard:ab
34. reliab*:ab
35. #1 OR #2 OR #3 OR #4 OR #5 OR #6 OR #7 OR #8 OR #9 OR #10 OR #11 OR #12 OR #13 OR #14 OR #15 OR #16 OR #17 OR #18 OR #19 OR #20 OR #21 OR #22 OR #23 OR #24 OR #25 OR #26 OR #27 OR #28 OR #29 OR #30 OR #31 OR #32 OR #33 OR #34
36. 'depression'/exp/dm_di,dm_pc
37. dysthymia:ti,ab,kw
38. blues:ti,ab,kw
39. melanchol*:ti,ab,kw
40. #36 OR #37 OR #38 OR #39
41. EPDS:ti,ab,kw
42. ‘Edinburgh Postnatal Depression’:ti,ab,kw
43. ‘Edinburgh Depression Scale’:ti, ab,kw
44. #41 OR #42 OR #43
45. #44 AND [2020-2023]/py

**Database: APA PsycINFO** **<1806 to February 08, 2023>**

1. Diagnosis/
2. Medical Diagnosis/
3. Psychodiagnosis/
4. Misdiagnosis/
5. Screening/
6. Health Screening/
7. Screening Tests/
8. Prediction/
9. Cutting Scores/
10. Psychometrics/
11. Test Validity/
12. screen*.af.
13. predictive value*.af.
14. detect*.ti.
15. sensitiv*.ti.
16. valid*.ti.
17. revalid*.ti.
18. accura*.ti.
19. psychometric*.ti.
20. specificit*.ab.
21. cut?off*.ab.
22. cut* score*.ab.
23. cut?point*.ab.
24. threshold score*.ab.
25. reference standard*.ab.
26. reference test*.ab.
27. index test*.ab.
28. gold standard.ab.
29. 1 or 2 or 3 or 4 or 5 or 6 or 7 or 8 or 9 or 10 or 11 or 12 or 13 or 14 or 15 or 16 or 17 or 18 or 19 or 20 or 21 or 22 or 23 or 24 or 25 or 26 or 27 or 28
30. exp "Depression (Emotion)"/
31. dysthym*.tw.
32. blues.tw.
33. melanchol*.tw.
34. 30 or 31 or 32 or 33
35. EPDS.af.
36. Edinburgh Postnatal Depression.af.
37. Edinburgh Depression Scale.af
38. 35 or 36 or 37
39. 29 and 34 and 38
40. limit 39 to yr="2020 -Current"

**Supplementary D. QUADAS-2 coding manual for original studies included in the present study**

Here, we referred to the QUADAS-2 instructions and the coding of a study (expect domain2: index test because our participant data was not tested at all cut-offs)^26,27^. We made no additions or subtractions to the QUADAS-2 problem.

**Domain 1: Participant Selection**

**1. Signaling question 1 – Was a consecutive or random sample of patients enrolled?**

Code as “yes” if a consecutive or random sample of participants were recruited for the study and the percentage of eligible participants who participate is ≥75%. If the study indicates that consecutive or random participants were recruited, but does not give an indication of the total number of eligible participants and how many agreed to participate in the study, this should be rated “unclear”. If the percentage of eligible participants included in the study was between ≥50% and ﹤75%, then this should also be marked as “unclear”. If a very low rate of eligible participants (﹤50%) were included in the study, this should be coded “no.” In “Notes”, please provide the relevant numbers and percentages used to make a determination. If a convenience sample of participants was recruited for the study or if the study was a case-control design, code as “no”.

**2. Signaling question 2 – Was a case-control design avoided?**

Code as “yes” if the study did not employ a case-control design. Code as “no” if the study used a case-control design.

**3. Signaling question 3 – Did the study avoid inappropriate exclusions?**

Inappropriate exclusions refer to situations where an important part of the screening population was excluded from the study based on characteristics that could be related to screening results. Code as “yes” if the study does not inappropriately exclude participants. Code as “no” if the study inappropriately excludes participants.

**4. Overall risk of bias**

Rate as “low”, “High”, or “unclear” as described in QUADAS-2. Please indicate factors in decision in “Notes”. NOTE: if signaling question 1 was coded “Unclear” the overall risk of bias is either a) Unclear, in cases where the denominator is not specified, or the percentage cannot be calculated, or method of participant selection is unclear OR b) Low, in cases where the percentage can be calculated, and is between 50- 75%. If signaling question 1 is a “no” and signaling questions 2 and 3 are both “yes” then the risk of bias is coded “Unclear”.

**5. Applicability concerns**

Code as “low” if study excluded participants who were already diagnosed or treated for depression or if the study included these patients, but they can be excluded using the individual patient data. Also code as “low” if the study did not exclude participants already diagnosed with depression and the overall percentage of these participants is low (e.g., ≤ 2.0% of total participants), even if there is not a variable to exclude them. Code “unclear” if the study did not exclude participants already diagnosed or treated for depression and it is not known how many diagnosed and treated patients were included or if the percentage is moderate (e.g., >2.0% but ≤ 5.0%). Code “High” if already diagnosed and treated patients are included and make up > 5.0% of the total sample and there is not a variable to exclude them. Please see aggregated study information sheet to code this.

**Domain 2: Index Test**

**1. Signaling question 1 - Were the index test results interpreted without the knowledge of the results of the reference standard?**

Code as “yes” if the index test is always conducted and interpreted before the reference standard. Code as “Unclear” if the study does not indicate the correlation between index tests and reference standards. Code as “no” if the index test is conducted and interpreted after the reference standard.

**2. Signaling question 2 - If a threshold was used, was it pre-specified?**

Code as “yes” if the study used a pre-specified threshold. Code as “Unclear” if the study does not use a pre-specified threshold or the study doesn't have any more information. Code as “no” if the study tested at all thresholds/cut-offs.

**3. Overall risk of bias**

Code as “low” if studies since the interpretation of the index test is fully automated in scoring self-report depressive symptom questionnaires and the individual participant data allows for testing at all thresholds/cut-offs.

**4. Applicability concerns**

Code “low” if the standard language version of the index test was used or if a translated version was used with an appropriate translation and back-translation process, or a translated version is located online. Code “unclear” if a translated version was used and it is not clear what steps were taken to ensure the quality of the translation or if only forward translation was used.

**Domain 3: Reference Standard**

**1. Signaling question 1 – Is the reference standard likely to correctly classify the condition?**

This question will be coded as “yes” for all studies because the use of a validated semi- or fully-structured psychiatric interview to assess participants for a DSM or ICD diagnosis of DD/DE is an eligibility requirement.

**2. Signaling question 2 – Were the reference standard results interpreted without knowledge of the results of the index test?**

Code as “yes” if the person administering the diagnostic interview was blinded to the participant’s score on the index test, or if the diagnostic interview was administered before the index test. Code as “no” if the person administering the diagnostic interview was not blinded or was aware of the participant’s score on the index test. Code as “unclear” if the study does not indicate whether blinding occurred and we cannot ascertain whether blinding occurred.

**Fully structured:** CIDI, DIS, CIS-R

**Semi-structured:** SCID, CIS

**MINI**

**3. Overall risk of bias**

The coding of this item should consider blinding of the person administering the diagnostic interview to the participant’s score on the index test.

**4. Applicability concerns**

This item will be coded as “low” for most standard language studies, since the use of a validated semi- or fully structured psychiatric interview to assess participants for a DSM or ICD diagnosis of DD/DE is an eligibility requirement. For translated versions of a validated reference standard, code “low” if a translated version was used with an appropriate translation and back-translation process, or a translated version is located online. Code “unclear” if a translated version was used and it is not clear what steps were taken to ensure the quality of the translation or if only forward translation was used.

**Domain 4: Flow and Timing**

**1. Signaling question 1 – Was there an appropriate interval between index test and reference standard?**

Only patient data with two weeks or less between the index text and reference standard are included. Thus, code “yes” if index test and reference standard were administered within a week of each other. Code “unclear” if the period was greater than one week (but less than two weeks) or if the timing cannot be ascertained beyond knowing that it was < 2 weeks. Note that this item may be coded differently for different patients from the same study. Please see aggregated study information sheet to code this.

**2. Signaling question 2 – Did all patients receive the same reference standard?**

This question will typically be coded as “yes” for all studies, since the reference standard is almost always consistent within each study.

**3. Signaling question 3 – Were all patients included in the analysis?**

When coding for this question, compare the number of participants who received the index test to the number of participants who received the reference standard. Code as “yes” if at least 90% of participants who received the index test also received the reference standard, or vice versa, and were included in analyses. Code as “unclear” if this difference is ≥ 80%, but < 90% or if it cannot be determined. Code as “no” if it is < 80%. If the study used randomly selected patients for either the index test or the reference standard, do not count the participants who did not receive the reference standard for that reason as missing.

**4. Overall risk of bias**

Rate as “low”, “High”, or “unclear” risk of bias.

If a study is judged as “low” on all domains relating to bias or applicability, then it is appropriate to have an overall judgment of “low risk of bias” or “low concern regarding applicability” for that study. If a study is judged “high” or “unclear” in 1 or more domains, then it may be judged “at risk of bias” or as having “concerns regarding applicability.”

**Supplementary E. The processing of raw data**

The study found that 81 participants were diagnosed with DE through the diagnostic interview out of the 560 people who underwent the two steps of PHQ-9 screening and diagnostic interview. We retrieved true positive, false positive, true negative, and false negative values for the cut-off scores of PHQ-9 from 5 to 15 based on the raw data offered in this study, and we computed the sensitivity and specificity of the PHQ-9 cut-off scores. The definition stated that 11 was the optimal cut-off score.

| cut-off score | Ture Positive | False Positive | Ture Negative | False Negative | Sensitivity | Specificity | Youden index |
| --- | --- | --- | --- | --- | --- | --- | --- |
| 5 | 77 | 389 | 90 | 4 | 0.95 | 0.19 | 0.14 |
| 6 | 76 | 335 | 144 | 5 | 0.94 | 0.3 | 0.24 |
| 7 | 72 | 300 | 179 | 9 | 0.89 | 0.37 | 0.26 |
| 8 | 72 | 261 | 218 | 9 | 0.89 | 0.46 | 0.35 |
| 9 | 69 | 225 | 254 | 12 | 0.85 | 0.53 | 0.38 |
| 10 | 67 | 188 | 291 | 14 | 0.83 | 0.61 | 0.44 |
| **11** | **63** | **156** | **323** | **18** | **0.78** | **0.67** | **0.45** |
| 12 | 55 | 132 | 347 | 26 | 0.68 | 0.72 | 0.40 |
| 13 | 54 | 110 | 369 | 27 | 0.67 | 0.77 | 0.44 |
| 14 | 44 | 92 | 387 | 37 | 0.54 | 0.81 | 0.35 |
| 15 | 41 | 69 | 410 | 40 | 0.51 | 0.86 | 0.37 |

**Supplementary F.** **QUADAS-2 ratings for each included original study**

|  | Domain 1: Participant Selection | | | | | Domain 2: Index Test | | | | Domain 3: Reference Standard | | | | Domain 4: Flow and Timing | | | |
| --- | --- | --- | --- | --- | --- | --- | --- | --- | --- | --- | --- | --- | --- | --- | --- | --- | --- |
| First Author, Year | SQ1 | SQ2 | SQ3 | RoB | AC | SQ1 | SQ2 | RoB | AC | SQ1 | SQ2 | RoB | AC | SQ1 | SQ2 | SQ3 | RoB |
| Tamrchi, 2021 | Yes | Yes | Yes | Low | Low | Yes | U/C | U/C | U/C | Yes | Yes | Low | Low | Yes | Yes | Yes | Low |
| Dering, 2022 | Yes | Yes | Yes | Low | Low | U/C | U/C | U/C | U/C | Yes | U/C | U/C | Low | Yes | Yes | Yes | Low |
| Pranckeviciene, 2022 | Yes | Yes | Yes | Low | Low | Yes | No | Low | Low | Yes | Yes | Low | Low | Yes | Yes | Yes | Low |
| Abdullah, 2022 | Yes | Yes | Yes | Low | Low | No | U/C | High | U/C | Yes | Yes | Low | Low | Yes | Yes | Yes | Low |

Abbreviations: AC: acceptability concern, RoB: risk of bias, SQ: signaling question, N/A: not applicable; U/C: Unclear

**Reference**

1. Screening for suicide risk in adolescents, adults, and older adults in primary care: recommendations from the U.S. Preventive Services Task Force. *Annals of internal medicine.* 2014;160(10):I-22.

2. Siu AL. *Screening for depression in adults: US Preventive Services Task Force recommendation statement.*: JAMA: Journal of the American Medical Association. Vol.315,(4), 2016, pp. 380-387.; 2016.

3. Siu AL, Bibbins-Domingo K, Grossman DC, et al. Screening for depression in children and adolescents: U.S. Preventive services task force recommendation statement. *Annals of Internal Medicine.* 2016;164(5):360-366.

4. Kransdorf LN, Files JA. *Management of depression in women.*: Journal of Women's Health. Vol.28,(8), 2019, pp. 1019-1022.; 2019.

5. Hess CW, Karter J, Cosgrove L, Hayden L. Evidence-based practice: A comparison of International Clinical Practice Guidelines and current research on physical activity for mild to moderate depression. *Translational Behavioral Medicine.* 2019;9(4):703-710.

6. Zuckerbrot RA, Cheung A, Jensen PS, Stein REK, Laraque D. Guidelines for adolescent depression in primary care (GLAD-PC): Part I. Practice preparation, identification, assessment, and initial management. *Pediatrics.* 2018;141(3).

7. Campos-Outcalt D. Whom to screen for anxiety and depression: Updated USPSTF recommendations. *Journal of Family Practice.* 2022;71(10):423-425 and 434.

8. Rafferty J, Mattson G, Earls MF, Yogman MW. Incorporating Recognition and Management of Perinatal Depression Into Pediatric Practice. *Pediatrics.* 2019;143(1).

9. Mangione CM, Barry MJ, Nicholson WK, et al. Screening for Depression and Suicide Risk in Children and Adolescents: US Preventive Services Task Force Recommendation Statement. *JAMA.* 2022;328(15):1534-1542.

10. Webber E, Benedict J. Postpartum depression: A multi-disciplinary approach to screening, management and breastfeeding support. *Archives of psychiatric nursing.* 2019;33(3):284-289.

11. Depression in adults. *National Institute for Health and Care Excellence: Guidelines.* 2011.

12. Depression in children and young people: identification and management. In: *National Institute for Health and Care Excellence: Guidelines.* London: National Institute for Health and Care Excellence (NICE)

Copyright © NICE 2019.; 2019.

13. Depression in adults: treatment and management. In: *National Institute for Health and Care Excellence: Guidelines.* London: National Institute for Health and Care Excellence (NICE)

Copyright © NICE 2022.; 2022.

14. Sethuraman B, Thomas S, Srinivasan K. Contemporary management of unipolar depression in the perinatal period. *Expert Review of Neurotherapeutics.* 2021;21(6):643-656.

15. Austin MPV, Middleton P, Reilly NM, Highet NJ. Detection and management of mood disorders in the maternity setting: The australian clinical practice guidelines. *Women and Birth.* 2013;26(1):2-9.

16. Hirshler Y, Gemmill AW, Milgrom J. An Australian perspective on treating perinatal depression and anxiety: a brief review of efficacy and evidence-based practice in screening, psychosocial assessment and management. *Annali dell'Istituto superiore di sanita.* 2021;57(1):40-50.

17. Andersen BL, DeRubeis RJ, Berman BS, et al. Screening, assessment, and care of anxiety and depressive symptoms in adults with cancer: An American Society of Clinical Oncology guideline adaptation. *Journal of Clinical Oncology.* 2014;32(15):1605-1619.

18. Malhi GS, Bell E, Bassett D, et al. *The 2020 Royal Australian and New Zealand College of Psychiatrists clinical practice guidelines for mood disorders.*: Australian and New Zealand Journal of Psychiatry. Vol.55,(1), 2021, pp. 7-117.; 2021.

19. McQuaid JR, Buelt A, Capaldi V, et al. The Management of Major Depressive Disorder: Synopsis of the 2022 U.S. Department of Veterans Affairs and U.S. Department of Defense Clinical Practice Guideline. *Annals of Internal Medicine.* 2022;175(10):1440-1451.

20. Frost J, Rich RL, Robbins CW, et al. Depression following acute coronary syndrome events: Screening and treatment guidelines from the AAFP. *American Family Physician.* 2019;99(12):786A-786J.

21. Quittner AL, Abbott J, Georgiopoulos AM, et al. International Committee on Mental Health in Cystic Fibrosis: Cystic Fibrosis Foundation and European Cystic Fibrosis Society consensus statements for screening and treating depression and anxiety. *Thorax.* 2016;71(1):26-34.

22. Colquhoun DM, Bunker SJ, Clarke DM, et al. Screening, referral and treatment for depression in patients with coronary heart disease: A consensus statement from the national heart foundation of Australia. *Medical Journal of Australia.* 2013;198(9):1-7.

23. Maki PM, Kornstein SG, Joffe H, et al. Guidelines for the evaluation and treatment of perimenopausal depression: Summary and recommendations. *Journal of Women's Health.* 2019;28(2):117-134.

24. Mackner LM, Whitaker BN, Maddux MH, et al. Depression Screening in Pediatric Inflammatory Bowel Disease Clinics: Recommendations and a Toolkit for Implementation. *Journal of Pediatric Gastroenterology and Nutrition.* 2020;70(1):42-47.

25. Depression Assessment Instruments. American Psychological Association (APA). <https://www.apa.org/depression-guideline/assessment>. Published 2022. Accessed.

26. Whiting PF, Rutjes AW, Westwood ME, et al. QUADAS-2: a revised tool for the quality assessment of diagnostic accuracy studies. *Ann Intern Med.* 2011;155(8):529-536.

27. Wu Y, Levis B, Sun Y, et al. Accuracy of the Hospital Anxiety and Depression Scale Depression subscale (HADS-D) to screen for major depression: systematic review and individual participant data meta-analysis. *BMJ.* 2021;373:n972.
